# Optimizing Professional Practice Evaluation to Enable a Nonpunitive Learning Health System Approach to Peer Review

**DOI:** 10.1097/pq9.0000000000000375

**Published:** 2020-12-28

**Authors:** Christy I. Sandborg, Gary E. Hartman, Felice Su, Glyn Williams, Beate Teufe, Nina Wixson, David B. Larson, Lane F. Donnelly

**Affiliations:** From the * Department of Pediatrics, Stanford University, School of Medicine, Palo Alto, Calif.;; † Department of Surgery, Stanford University, School of Medicine, Palo Alto, Calif.;; ‡ Department of Anesthesia, Stanford University, School of Medicine, Palo Alto, Calif.;; § Center for Pediatric and Maternal Value, Lucile Packard Children’s Hospital, Stanford, Stanford Children’s Health, Palo Alto, Calif.; and; ¶ Department of Radiology, Stanford University, School of Medicine, Palo Alto, Calif.

## Abstract

Healthcare organizations are focused on 2 different and sometimes conflicting tasks; (1) accelerate the improvement of clinical care delivery and (2) collect provider-specific data to determine the competency of providers. We describe creating a process to meet both of these aims while maintaining a culture that fosters improvement and teamwork.

**Methods:**

We created a new process to sequester activities related to learning and improvement from those focused on individual provider performance. We describe this process, including data on the number and type of cases reviewed and survey results of the participant’s perception of the new process.

**Results:**

In the new model, professional practice evaluation committees evaluate events purely to identify system issues and human factors related to medical decision-making, resulting in actional improvements. There are separate and sequestered processes that evaluate concerns around an individual provider’s clinical competence or behavior. During the first 5 years of this process, 207 of 217 activities (99.5%) related to system issues rather than issues concerning individual provider competence or behavior. Participants perceived the new process as focused on identifying system errors (4.3/5), nonpunitive (4.2/5), an improvement (4.0/5), and helped with engagement in our system and contributed to wellness (4.0/5).

**Conclusion:**

We believe this sequestered approach has enabled us to achieve both the oversight mandates to ensure provider competence while enabling a learning health systems approach to build the cultural aspects of trust and teamwork that are essential to driving continuous improvement in our system of care.

## INTRODUCTION

Over the past 2 decades, 2 priorities are emphasized for healthcare organizations. One of those priorities is to create the culture and processes to accelerate the improvement of clinical care delivery.^1–3^ A second priority is to have processes that collect provider-specific data [Professional Practice Evaluation (PPE)] to determine providers’ competency to provide high-quality, safe patient care.^4–11^ These 2 priorities can be at odds culturally, as accelerating improvement requires a culture of safety, mutual trust, transparency, collaboration, and understanding that error identification is critical to identifying and addressing problems.

In contrast, evaluating individual provider competence is measured in 2 ways: (1) generic quality and safety metrics and (2) the traditional peer-review approach of judging the potential contributions of individual providers’ actions based on case reviews where harm and/or “near misses” have occurred. Providers can experience the latter process as judgmental and punitive, and often the antithesis of what we recognize as the best approach to error prevention—a culture of safety. When these 2 aims coexist in the same process, there may be an erosion of providers’ trust in the institution’s commitment to a culture of safety.^12–15^

### Monitoring the Competency of Providers

For healthcare organizations accredited by organizations such as The Joint Commission (TJC) and Centers for Medicare and Medicaid Services, there is an emphasis that “determining the competency of providers to provide high quality, safe patient care is one of the most important” activities in which they partake.^4,5^ TJC states that credentialing and privileging processes must involve “a series of activities designed to collect, verify, and evaluate data relevant to a provider’s professional performance” and that these data factor into significant decisions such as “recommendations to grant or deny initial and renewed privileges.”^4^ The data may also factor into decisions to limit or revoke the provider’s privileges.^6^

There are requirements for organizations to have defined processes to continuously evaluate a provider’s performance via Ongoing PPE (OPPE). ^4,6–9^ Guidelines for OPPE state that parameters for OPPE can be derived from peer review or peer recommendation.^7^ However, details of how organizations conduct peer-review processes are left to the individual organizations. TJC guidelines also require that improvement activities use information obtained during the OPPE process.^11^

Although the task of protecting patients from the potential of providers whose skills, ethics, or behaviors are lacking is a critical role for healthcare organizations, there are downsides to identify, coach and improve, or potentially remove outlier providers when the process relies on peer review.^12^ Providers will perceive processes that put a provider’s credentialing, privileging, and ability to make a living by practicing medicine at risk as potentially punitive. Such processes can undermine a culture of trust, and when such processes use peer-review data, they can compromise relationships between providers. Using a process such as traditional peer review that focuses on finding what is wrong with a provider’s practice negatively impacts error reporting, relationships between providers and team members, a high functioning team, and trust in the organization.^13–15^

### Fostering System Improvement in Clinical Care

There has been a movement in medicine, particularly in radiology, away from peer review to peer learning.^12–15,16–18^ Radiology has been moving from a traditional peer-review system in which the initial radiology report is reviewed by a second radiologist and the degree of error graded by a numerical scoring system.^19–21^ Error rates for individual radiologists are calculated. Those error rates could be used as a parameter in OPPE for radiologists.^12–15,16–21^ However, this process leads to under-reporting of errors, prevents the promotion of learning, and is not considered valuable by participating radiologists.^12–15,16–18,22–26^ Also, identifying an outlier, a poorly performing radiologist, via this process was exceedingly rare.^15^ The lack of value of this peer-review approach led to the development and implementation of peer learning, in which the goal is to learn and improve the delivery of care as a team.^13–15,16–18^ The critical steps in this transition are listed in Table 1.^13^ Experiences with this transition have shown increased reporting of errors.^15^ Radiologists perceive peer learning as nonpunitive, of greater value, and more conducive to learning and improvement than the previous peer-review–based system.^15^

**Table 1.** Key Steps in Transitioning from Peer Review to Peer Learning [15]

• Sequestering learning and improvement activities from those designed to monitor for deficient performance
• Moving from random sampling of cases to active inclusion of identified learning opportunities
• Replacing numerical scoring of errors with qualitative descriptions of learning opportunities
• Providing confidential and constructive feedback to providers
• Conducting effective peer learning conferences
• Linking the peer learning program to process improvement infrastructure

This article outlines these challenges and describes the structure and processes for the evolution of PPE for all specialties at one hospital through leveraging the radiology peer learning model. This approach permits maintaining a comprehensive nonpunitive response to error to foster a learning health system approach, based on the IOM definition of a learning health system in which alignment of scientific and cultural tools improves healthcare as a result of daily practice.^3^ Our focus is on the approach to PPE broadly rather than on the specific process of OPPE.

## METHODS

Peer learning concepts can help optimize the structure of an institutional, PPE process. It evaluates errors and near misses related to medical decision-making, providers’ roles, and systems issues. Our organization, a quaternary academic children’s hospital that manages approximately 360 beds, has embarked on changing our PPE process for providers. The goal was to change the structure to create a less punitive learning environment that focused on identified system issues, rather than finding the few individual outliers with competency or behavioral issues.

This initiative was led by the Care Improvement Committee (CIC), which oversees the PPE programs at our institution. A medical and surgical co-chair leads the CIC. Current membership includes leaders from each PPEC, team members from the quality and safety department, representatives from nursing leadership, and risk management. The institution’s clinical stakeholders include the chief quality officer, the chief medical officer, chief nursing officer, and the chief experience officer. CIC reports its activities to the Medical Executive Committee.

Five years ago, the CIC recognized that our traditional peer review process did not create an open learning environment that could address the complexity and the acuity of the cases reviewed. Some of the most concerning care gaps and failures in medical decision-making were due to challenges with communication, collaboration, and coordination when multiple subspecialty groups were involved. The previous peer-review process led to each subspecialty pointing fingers at the others and failing to collaborate in identifying learning opportunities. Within each preexisting subspecialty peer-review committee, peers were reluctant to attribute responsibility for an error to their colleagues. This reluctance was likely due to concerns about preserving professional relationships and the ongoing ability to work together.^2^

To address these issues, the CIC created a task force to design a better process going forward, with a primary focus on moving to a nonjudgmental approach to improvement similar to the radiology peer learning process. The results include a new process designed by that task force.

### The New Model

**Fig. 1.**
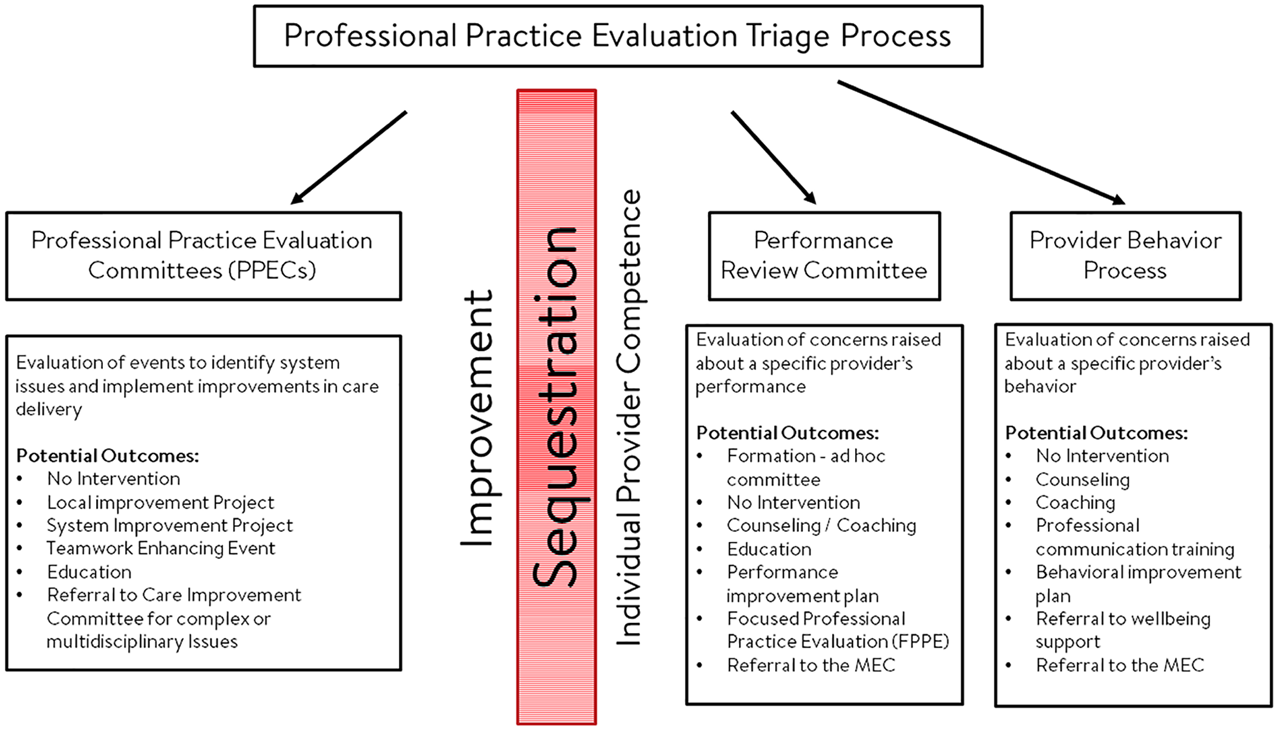
Diagram outlining the separation of the improvement from the individual provider competence portions of PPE. PPECs include Heart Center, General Pediatric and Medical Specialties, Pediatric Critical Care Medicine, Solid Organ Transplant, Surgical Specialties, Trauma, Obstetrics, Anesthesia, Neonatology, Pathology, and Radiology. MEC, Medical Executive Committee

The task force created a new model. It involved creating three committees: the subspecialty-oriented *PPECs*, the *Performance Review Committee* (PRC), and the *Provider Behavioral Process* (PBP; Fig. 1). The PRC and PBP are called on an *ad hoc* basis. The majority of participants in these ad hoc processes are members of the PPECs or CIC. The small number of cases concerning individual providers’ competency or behavior are sequestered from the PPEC process and redirected for in-depth review at the PRC or the PBP. This approach preserves a culture conducive to fostering improvement learning opportunities. This new process allowed several improvements, including a focus on collaboration with the safety and quality teams in implementing error prevention behaviors,^27^ root cause analyses for serious safety events, and a more in-depth focus on inter- and intra-professional teamwork.

### Professional Practice Evaluation Committees

**Fig. 2.**
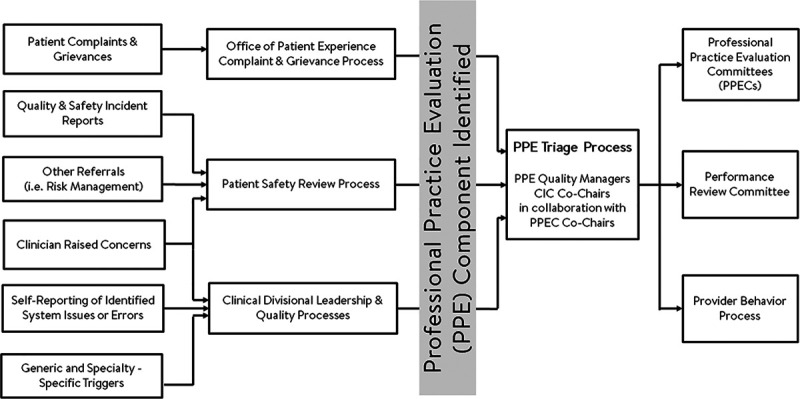
Diagram outlining the flow of information gained about cases through various sources for PPE.

The PPECs are subspecialty-oriented committees that review identified incidents to improve professional aspects of clinical care and medical decision-making. Cases are referred to the PPECs using various inputs, including prespecified triggers, incident reports, serious safety events, and patient complaints (Fig. 2). The PPECs review cases and identify improvement opportunities in both systems, human, and team factors domains. As mentioned previously, there is no direct attribution to individual providers in the PPEC process. Notably, a separate hospital event review process evaluates the approximately 8,000/year filed incident reports for events that have system-wide issues and evaluates the degree of harm. During that process, if events are identified with a professional-related issue primarily, they are referred for review at the appropriate PPEC.

Currently, there are 12 different subspecialty-oriented PPECs. Several of the PPECs include multiple provider subspecialties as well as interprofessional non-MD members for broader input. Each PPEC has 2 provider co-leaders, a supporting quality manager, provider members from each of the represented subspecialties, and relevant nursing or other clinical representatives. The PPEC process involves a significant percentage of our medical staff, with approximately 20% of clinically active providers involved, creating a robust learning community.

The PPECs recommend performance improvement plans to address the identified gaps (Fig. 1). Often, if the plan pertains to the PPECs specialty area(s), the improvement plan can be moved forward without resources external to that area. In those cases where the issues are multidisciplinary and interprofessional, the PPECs often refer to CIC to enable collaboration between stakeholders with the support of safety and quality teams to develop and implement a shared plan. PPECs also perform common cause analysis to identify themes that can result in broader system improvement proactively.

The opportunity to recognize and celebrate exemplary care is also possible with this approach to PPE case reviews. In some case analyses, PPEC members recognize that the robust efforts of highly effective care teams have averted the occurrence of a more serious harm outcome. Expressing gratitude for what has gone well is an essential human factor intervention that promotes well-being and fosters intrinsic motivation.^2,28^

### Performance Review Committee

Quality and safety leadership or PPEC co-chairs act to redirect any potential issues about individual performance to the PRC. In cases where a PPEC is reviewing system issues, and an individual performance issue is identified, the PPEC will continue to review the case. Still, reviewing the specific issues related to the individual performance issue will be transferred for review via a PRC, outside the PPEC process. We sequester this process from the PPEC process and allow a case-by-case in-depth review in collaboration with Medical Staff leadership (Fig. 1). Upon referral of a provider to the PPE program, the CIC co-chairs meet with the individual’s supervisor (chief or chair). The leadership of the Medical Staff (chief medical officer, chief quality officer , and elected President of the Medical Staff) to determine if a review should precede. If the review should proceed, an ad hoc committee is formed, including the Medical Staff leadership, (chief/chair) and subject matter experts (peers). The assembled PRC meets a minimum of three times: (1) to review the concerns about the provider in detail; (2) to meet with the provider to obtain their perspective; and (3) to deliberate regarding potential actions. A follow-up meeting with a subset of the ad hoc committee discusses the recommendations with the provider. Figure 1 lists potential actions.

### Provider Behavioral Process

Potential issues concerning an individual provider’s behavior that disrupts or undermines the culture of safety or a respectful workplace are referred to the PBP program. Such events are identified via several inputs, including incident reports or patient or co-worker complaints. This process is also sequestered from the PPEC process (Fig. 1). Interviews with concerned individuals by management staff validate the behavioral concern. A tiered algorithm is used to validate the behavioral incident (Fig. 1). The algorithm considers the frequency of events or seriousness of the behavior’s impact on the workplace.

All deliberations surrounding the three committees and processes are confidential and protected under relevant peer review state law. However, to support the culture of safety, ongoing education of staff and providers regarding the general approach to ensuring the quality, competence, and professional behavior of providers on the Medical Staff is conducted regularly. Figure 1 lists potential outcomes for each workflow.

In cases where a PPEC is reviewing system issues, and an individual behavioral issue is identified, the PPEC will continue to review the case. The review of the behavioral issues related to the individual performance issue will be transferred for review via a PBP, outside the PPEC process.

### Ongoing Professional Practice Evaluation

Although this manuscript intends to describe our overarching approach to PPE, we thought that including a brief description of our OPPE program by highlighting touchpoints between OPPE and our PPE program was pertinent. Our OPPE program is designed to meet the requirements of TJC.^4–11^ We track provider-specific parameters that cover all six of the Accreditation Council for Graduate Medical Education general competencies.^4–11^ Some of the OPPE metrics are generalizable to all providers. Such parameters include items such as attestation to meeting annual educational requirements, timely completion of medical records, and avoiding medical record suspension. One general parameter does relate to PPEC activities: examples of exemplary care and the number of patient and family grievances (goal *<* one) can be added to the physician’s OPPE file. These are typically identified via the PPEC case. The number of behavioral events (goal *<* one) is also recorded from PBP activity. In addition to these general parameters, each clinical area has specialty-specific parameters chosen and data collected by the clinical chief. The nature of these specialty-specific parameters varies depending upon the specialty in question.

### Provider Satisfaction Survey

A 4-question survey was created, and an email with a link to the survey sent to all participants in PPECs and CIC members to address changes in the PPE over the past 5 years. The email stated that the survey was an evaluation of the entire PPE process. A five-point Likert scale was used with 1 = strongly disagree, 2 = disagree, 3 = neither agree or disagree, 4 = agree, and 5 = strongly agree. The results include the percentage of those surveyed who participated and provided answers to the questions. The 4 questions were as follows:

1. The PPEC Process is focused on identifying system errors.
2. The PPEC Process is nonpunitive.
3. Over the past 5 years, the PPEC process changes have been an improvement compared to the historical processes.
4. My participation in the PPEC process makes me feel more engaged in our clinical care system and contributes to my well-being.

We calculated the mean ranking for each question.

## RESULTS

### Committee Activity

Table 2 summarizes numerical data concerning the activities of the PPECs. The mean number of cases reviewed annually by the PPECs was 207. The mean number of cases annually referred from a PPEC to CIC because of multidisciplinary issues was 22. The mean number of local improvement plans implemented within PPECs annually was 29. Two to three of these improvement plans annually are complex multispecialty projects. Common cause analysis to evaluate significant system issues has led to four occasions’ organizational action plans in the past two years.

**Table 2.** Summary of Activities Occurring in PPECs

Year	PPEC Activities		
	Cases Reviewed*	CIC referrals	Local Performance Improvement Plans
2016	201	21	32
2017	238	20	31
2018	187	24	30
2019	203	21	23
Mean	207	22	29

On average, there are 2–4 PRC reviews annually. Likewise, on average, there are approximately 7 PBP reviews annually. Therefore, the annual percentage of PPE activities related to individual practitioner competence or behavior is 10 of 217 or 0. 5%. The overwhelming number of activities (99.5%) in PPE annually is related to system issues evaluated by PPECs.

### Survey Results

We sent a link to the survey to 142 participants in the PPEC and CIC processes. One hundred four participants (73.2%) participated in the survey—Table 3 summarizes survey results. Participants tended to agree that the PPE process was focused on identifying system errors (4.3/5), nonpunitive (4.2/5), an improvement (4.0/5), and helped with engagement in our system and contributed to wellness (4.0/5).

**Table 3.** Survey Results of Participants’ Perception of the PPE Process

Question	1 Strongly Disagree	2 Disagree	3 Neutral	4 Agree	5 Strongly Agree	Number Responses	Mean Score
The PPEC process is focused on identifying system errors	1	0	10	51	41	103	4.3
The PPEC process is nonpunitive	0	0	15	49	39	103	4.2
Over the past 5 years, the changes made to the PPEC process have been an improvement as compared to the historic processes	0	5	23	42	31	101	4.0
My participation in the PPEC process makes me feel more engaged in our clinical system of care and contributes to my well-being	1	6	20	38	39	104	4.0

## DISCUSSION

Having a single process that attempts to identify poor performing providers and foster and accelerate improvement in clinical care delivery can have significant cultural challenges that can limit the success of achieving either of these aims. We have designed our system for PPE to have separate processes so that both accelerated learning and improvement, as well as addressing individual provider competence or behavior, can be accomplished. The process relies on a series of subspecialty PPECs that focus on reviewing events solely to identify systems and human factors issues and to create and implement performance improvement plans targeting sustainable improvement. The events reviewed by PPECs represent 99.5% of cases referred for potential harm or near misses.

The much less frequent instances of potential individual provider clinical competence or behavioral issues are not reviewed in the PPECs, by design, to optimize a nonpunitive response to error critical to a healthy culture of safety and continuous learning health systems. We sequester the PRC processes for competence concerns and the PBP for concerns about provider behavior from work aimed at the improvement of professional services. These activities are primarily managed by the medical staff leadership and the individual provider’s supervisor (chief/chair). We believe that the separation of these processes has enabled us to achieve both the oversight mandates to ensure provider competence while at the same time maintaining the cultural aspects of trust and teamwork that are essential to driving improvement in our system of care.

A change to nonjudgmental language to identify potential contributors to harm or near misses also enhanced the new nonpunitive approach. The older analytic framework included phrases such as “error in diagnosis,” “error in technique,” or “error in judgment,” which heightened the punitive nature of these reviews. Opportunities to nurture teamwork, grow skills, refine systems, and support well-being were neglected. To enhance learning and improvement opportunities that arise from the PPEC case reviews, a new causal analysis framework to evaluate the events, based on the taxonomy of errors created by Healthcare Performance Improvement^29^ has been rolled out in the PPECs over the past 4 years. This change has facilitated the identification of actionable improvements in the area of human factors or system issues.

This review of our process has several limitations. First, our approach to having multiple PPECS performing case-based reviews in addition to our hospital event review process is unique and perhaps not easily transferrable to other systems. Second, although our survey was sent out to get participants’ perception of our overall approach to PPE—including the PPEC, PBP, PRC, and OPPE components—it is not completely clear what the participants perceive as nonpunitive. Is it just PPEC or the whole approach? Finally, although over the past several years, we have seen a dramatic improvement in many of our quality and safety indicators,^30,31^ we do not have data that proves that all of these efforts in PPE have contributed directly to improvement in our services. Subjectively, we do think this work has added value.

## DISCLOSURE

The authors have no financial interest to declare in relation to the content of this article.
